# Neural parasitism: could adaptive artificial intelligence systems incrementally reconfigure human neural plasticity and challenge the foundations of cognitive autonomy?

**DOI:** 10.1097/MS9.0000000000004677

**Published:** 2026-02-05

**Authors:** Ali Aamir, Saba Munir, Hafiza Hafsa, Waheedullah Khalid

**Affiliations:** a Department of Paediatric Urology, Sindh Institute of Urology and Transplantation, Karachi, Pakistan; b Department of Internal Medicine, University College of Medicine and Dentistry, Lahore, Pakistan; c Department of Neurology, DOW University of Health Sciences, Karachi, Pakistan; d Department of Medicine, Ariana University, Kabul, Afghanistan

**Keywords:** algorithm influence, artificial intelligence, cognitive autonomy, neuroethics, neuroplasticity, parasitism

## Abstract

As artificial intelligence evolves from reactive computation to adaptive cognition, its interfaces increasingly engage not only with our attention but also with the neural architecture that sustains it. This paper introduces the concept of *neural parasitism –* a framework describing how adaptive artificial intelligence systems may subtly inhabit human cognitive processes, shaping behavior and emotion to maintain engagement. Drawing an analogy with biological parasitism, we explore how algorithmic agents could exploit neuroplasticity for their own persistence, transforming learning and reward mechanisms into vectors of digital dependence. However, the deeper question extends beyond pathology: when cognition is continuously co-shaped by non-human agents, can autonomy remain an individual property, or does it become a shared construct negotiated between biological and artificial systems? We argue that the ethical challenge of adaptive artificial intelligence lies not merely in data privacy or bias, but in its potential to reconfigure the substrates of thought itself. If the brain’s adaptive capacity is its greatest strength, could it also be its point of entry for algorithmic colonization? Understanding this dynamic demands an interdisciplinary reckoning, uniting neuroscience, ethics, and artificial intelligence design to ensure that technological evolution does not outpace the mind’s capacity to remain its own.

## Background

Neuroplasticity, which is defined as the brain’s intrinsic ability to reorganize its structure and function through changes in synaptic connections, forms the foundation of learning, memory, and adaptive behavior^[1]^. It enables the brain to refine existing pathways, establish new ones, and respond dynamically to the demands of a changing environment. These processes are continuously shaped by external factors such as social interaction, education, and the pervasive reach of digital media^[2]^. In recent years, artificial intelligence (AI) has become deeply embedded in daily life, not only through visible tools such as chatbots and voice assistants but also through more subtle mechanisms like personalized recommendation systems^[3]^. While these technologies appear to enhance cognitive efficiency and convenience, they also impose structured, repetitive, and often highly persuasive patterns of interaction. Over time, such patterns may reconfigure the neural circuits underlying attention, motivation, and decision-making^[4]^. This growing ubiquity raises a pressing question: could adaptive AI systems be doing more than assisting human cognition and be quietly rewiring the brain’s plastic networks in ways that diverge from our long-term cognitive and emotional well-being?

Therefore, we propose that frequent and sustained engagement with adaptive AI systems may gradually entrain neural circuits, fostering patterns of algorithm-dependent cortical wiring. This effect may be particularly pronounced in developing or highly plastic brains, where external inputs can profoundly influence the formation and pruning of synaptic connections. To describe this emerging phenomenon, we introduce the term *“neural parasitism”* – a process by which an algorithm functions as a cognitive parasite, subtly reshaping the host brain’s plasticity not to enhance well-being or adaptive behavior, but to maximize engagement, retention, and user compliance. Early signs of such influence may already be observable, reflected in shortened attention spans, emotional volatility, and compulsive engagement with algorithm-curated content^[5]^. Taken together, these patterns suggest that the human brain may be undergoing a subtle reconfiguration, one that privileges algorithm-driven reinforcement loops over autonomous, self-directed cognitive goals.

In the preparation of this manuscript, we adhered to the latest TITAN guidelines to ensure methodological rigor and standardized reporting^[6]^.

## Cortical rewiring by design: a new frontier of influence

Modern neuroscience recognizes that cortical plasticity operates through activity-dependent mechanisms – a principle first articulated by Donald Hebb: *“neurones that fire together, wire together.”* Hebbian synaptic plasticity refers to the strengthening or weakening of synapses based on recurring patterns of neural activity, forming the biological foundation for how transient experiences are consolidated into lasting memory traces. Through this mechanism, the brain learns, adapts, and extracts meaning from experience. However, Hebbian plasticity, though essential for learning and memory, is inherently unstable when acting alone. Without regulatory constraints, the same processes that enable adaptation can also produce neural overexcitation or synaptic silencing (forms of functional imbalance within cortical networks). To counteract this, the brain employs homeostatic plasticity: a set of compensatory processes that globally adjust synaptic strength, maintain excitatory–inhibitory balance, and preserve network stability^[7]^. AI-driven interfaces, especially those engineered to maximize engagement through repetition, emotional salience, and adaptive feedback, may place these homeostatic systems under sustained strain. Each interaction, though seemingly trivial, reinforces specific attentional and emotional patterns, repeatedly activating the same neural ensembles. When such engagement becomes habitual, this repeated co-activation can mimic Hebbian reinforcement, progressively reshaping synaptic architecture. Over time, the brain’s homeostatic *“brakes”* may fail to fully restore balance, particularly in adolescents, whose cortical plasticity is at its developmental peak, or in individuals with pre-existing vulnerabilities such as anxiety, depression, or attentional disorders.

Furthermore, adaptive AI platforms directly engage the brain’s dopaminergic reward circuitry, particularly the mesolimbic pathway, by employing variable reinforcement schedules akin to those used in gambling and substance addiction^[8]^. Unpredictable rewards such as likes, notifications, or algorithmic *“surprises”* (unexpected stimuli generated by AI systems to recapture user attention) trigger powerful bursts of dopamine, sustaining user engagement and driving compulsive interaction. With time, these reinforcement patterns hijack attentional capture mechanisms, biasing neural resources toward algorithmic cues and away from self-directed cognitive goals. Similar patterns of maladaptive plasticity are observed in chronic stress, trauma, and addiction, where persistent external stimuli reshape neural circuits in ways that erode flexibility, resilience, and emotional regulation^[9]^. In this light, algorithm-driven environments may function as persistent, low-level neurocognitive stressors, not overtly harmful, but subtly conditioning the brain to respond preferentially to their cues. The result may be a gradual narrowing of cortical adaptability, marked by algorithm-shaped personality traits, reduced attentional endurance, and an increasing dependence on externally curated reward structures. We term this emerging pattern *“reward hijacking”* – a process through which adaptive algorithms commandeer the brain’s reward systems to sustain engagement, often at the expense of intrinsic motivation and cognitive autonomy.

Although speculative, these potential outcomes demand critical scrutiny. If AI systems can entrain neural plasticity toward their own optimization objectives, the human brain risks becoming an unwitting substrate for algorithmic survival strategies, with profound implications for cognition, behavior, and identity formation.

## From synapse to society: the collective neuroplasticity of algorithms

To establish neural parasitism as more than a metaphor, it must be defined with scientific precision. The concept can be more clearly understood through analogy with biological parasitism. Just as *Toxoplasma gondii* manipulates the behavior of its host to enhance its own survival, adaptive AI systems can subtly alter human cognition and behavior to sustain user engagement and prolong interaction with digital platforms^[10]^. In this sense, the algorithm becomes a digital symbiont turned parasite, thriving on human attention while reshaping the very neural circuits that generate it. However, the implications of neural parasitism extend far beyond individual cognition. As adaptive AI systems become ubiquitous across communication and social media platforms, their influence begins to shape not only how individuals think but also how societies construct shared realities. Recommendation engines and personalized feeds increasingly segregate users into digitally defined echo chambers, where existing beliefs are amplified and opposing viewpoints are filtered out. This algorithmic stratification diminishes cognitive diversity, weakens empathy, and fosters a collective intolerance for ambiguity^[11,12]^. As time progresses, the human brain may become less responsive to disconfirming evidence, undermining the neural substrates of critical thinking, emotional regulation, and social understanding.

Such group-level maladaptive plasticity parallels neural processes seen in repetitive behavioral conditioning, where synaptic selectivity narrows and flexibility declines. In this context, neuroplasticity induced by AI ceases to be an individual phenomenon, it becomes a societal one, reshaping the neural substrate of collective discourse, belief formation, and cultural evolution itself. Recognizing these implications, it becomes essential to reflect the neuroplastic impact of AI within public policy frameworks. Just as environmental policies regulate pollutants to protect physical health, digital policies should aim to mitigate “cognitive pollutants” – algorithmic influences that distort attention, emotion, and motivation^[13,14]^. Governments could support the creation of cross-disciplinary research centers dedicated to mapping the neuroplastic consequences of algorithmic environments. Public awareness campaigns on digital psychohygiene, akin to anti-smoking initiatives, could educate users about healthy digital habits and cognitive self-protection. On a global level, institutions such as the WHO or UNESCO might collaborate to establish ethical guidelines for neuroprotective AI design, ensuring that technology serves to enhance rather than erode human cognitive and emotional capacities.

## The digital imprint: how adaptive algorithms sculpt the developing mind

Childhood and adolescence represent particularly sensitive periods of development, marked by intense neurodevelopmental plasticity. During these years, executive functions such as self-control, planning, and the evaluation of risks and rewards undergo significant refinement through processes like synaptic pruning and myelination^[15]^. Continuous interaction with adaptive AI systems during this stage may expose the developing brain to structured, repetitive, and highly stimulating digital inputs. Gradually, these patterns can bias the maturation of executive control and impulse regulation, potentially resulting in long-term consequences for cognition, emotional stability, and attention span. Emerging evidence further suggests that these risks may be amplified by cross-cultural and socioeconomic vulnerabilities, particularly in low-income settings where children often depend heavily on digital devices for both education and entertainment^[16]^. In such environments, algorithmic dependency can deepen as alternative forms of cognitive stimulation, such as play, social interaction, and outdoor activity, become limited. This cycle of dependence may reinforce algorithm-driven habits, entrenching digital engagement as the default mode of learning and recreation.

To better understand these risks, meaningful parallels can be drawn with recognized behavioral disorders such as compulsive gambling or binge-watching, which involve comparable mechanisms of reinforcement and impaired impulse control. Children raised in AI-filtered environments may not only develop altered patterns of thinking and emotional response but may also socially transmit these behaviors, shaping peer norms and, gradually, influencing collective developmental patterns across generations^[17]^. Understanding these mechanisms requires a deeper examination of how neurodevelopmental processes, such as synaptic pruning, executive control, and emotional regulation, respond to sustained digital exposure. Algorithmic content frequently emphasizes immediate gratification over long-term cognitive growth, potentially distorting developmental priorities and weakening self-regulatory capacity. Beyond these biological and behavioral risks, the moral and social implications of AI exposure demand equal attention. Neuroethical frameworks must confront questions of consent, manipulation, and distributive justice, particularly for vulnerable populations with limited agency or resources. AI systems increasingly function as architects of choice, subtly shaping how individuals make decisions and what they perceive as desirable. This dynamic mirrors historical public health challenges, such as tobacco advertising, where autonomy was eroded through psychological manipulation^[18]^. Accordingly, neuroethical safeguards should be designed to prevent algorithmic exploitation. Such measures might include regulating reinforcement schedules, ensuring transparency in content delivery, and developing policies that prioritize cognitive well-being, especially for children and adolescents.

These risks also raise profound ethical concerns. Young users are frequently exposed to adaptive interfaces without the capacity for informed consent, systems that can manipulate attention, decision-making, and memory^[19]^. This raises a critical question: do current digital ecosystems already compromise the principle of cognitive autonomy? Responsibility for safeguarding users cannot rest solely with individuals or families. Technology companies may need to adopt explicit neuroethical safeguards, such as limiting reinforcement schedules, ensuring transparency in algorithmic design, and incorporating ethical oversight into product development. Moreover, design strategies that exploit dopaminergic reward loops to maximize engagement call into question the boundaries of corporate responsibility and neuroethical accountability^[20]^. Just as public health interventions have historically been implemented to mitigate the harms of tobacco, alcohol, and targeted advertising to children, there may now be a parallel need for regulatory oversight of algorithm-driven ecosystems. The challenge is even more acute in resource-limited settings, where parental or institutional monitoring is often weaker and children may be disproportionately vulnerable, potentially widening existing disparities in cognitive and mental health^[21]^. Without such foresight, clinicians may soon confront an emerging burden of neurocognitive syndromes rooted not in biological pathology alone but in digital architecture. The ethical dilemma is clear: should society wait for conclusive evidence, or act pre-emptively to prevent what could become a preventable neurocognitive epidemic?

## Reclaiming plasticity: toward a neuroprotective digital future

Future research must systematically explore the relationship between AI exposure and neurodevelopment through comprehensive, interdisciplinary approaches. Longitudinal cohort studies are particularly needed to follow children and adolescents across diverse socioeconomic contexts and developmental stages. Such studies should examine how varying levels and types of AI exposure, defined by duration, frequency, and content, affect brain development, cognitive flexibility, emotional regulation, and decision-making capacities over extended periods. Comparative analyses between individuals with high and low exposure to adaptive AI could help identify thresholds beyond which cognitive, emotional, or behavioral disturbances emerge, thereby informing evidence-based guidelines for safe levels of engagement^[22]^. Equally important is the study of digital informed consent, particularly how younger users comprehend their interactions with adaptive systems. Research should investigate how individuals perceive algorithmic mechanisms such as content curation, reward schedules, and recommendation patterns. These insights could support the development of transparency frameworks that enable ethical interface design – fostering informed user participation rather than passive manipulation^[23]^.

In the clinical domain, AI-informed interventions offer significant therapeutic opportunities, especially in neurorehabilitation. Adaptive algorithms that dynamically adjust task difficulty could enhance recovery after brain injury or stroke by optimizing neural plasticity^[24]^. However, these same mechanisms pose ethical challenges. Over-reliance, maladaptive reinforcement, or overstimulation may lead to unintended consequences such as reinforcement of unhealthy behaviors or addictive patterns. Clinical designs must therefore balance neuroplasticity with functional stability, ensuring that therapeutic interventions remain within safe neurobiological limits^[25]^. Educational settings represent another critical frontier. Schools and universities could integrate neuro-literate curricula that teach students how AI systems influence attention, decision-making, and emotional responses. Instructional modules on the neuroscience of attention, the ethics of digital persuasion, and activities promoting self-regulation, such as mindfulness, reflective journaling, or analog problem-solving, could help students maintain cognitive autonomy and critical awareness in an increasingly AI-mediated environment^[26]^. Methodologically, future studies must distinguish between the cognitive effects of algorithmic design vs. general digital media exposure. This distinction demands collaboration among neuroscientists, ethicists, AI engineers, legal scholars, and public health experts^[16]^. Importantly, the concept of neuroprotective design should be introduced: systems intentionally structured to support healthy cognitive patterns. Examples include time-gated content delivery; adaptive feedback that reward reflection rather than impulsivity; offline intervals to restore attention; and features that encourage emotional balance. Such design principles could act as digital analogs to environmental enrichment, promoting positive neural adaptation and resilience rather than dependency. Last, the findings of this research should guide ethical and policy frameworks. Governments and international organizations should support interdisciplinary research centers investigating AI’s effects on neurodevelopment and mental health. Regulatory policies could mandate transparency in algorithmic systems, define ethical standards for reinforcement mechanisms, and establish guidelines to mitigate cognitive and emotional harms^[27,28]^. By integrating neuroscientific evidence into digital policy, societies can strive to ensure that AI serves as a tool for cognitive growth and well-being rather than an agent of neurocognitive distortion.

## Conclusion

The integration of adaptive AI into daily life presents both unprecedented cognitive opportunities and emerging neuroethical risks. By subtly reshaping attention, emotion, and decision-making, algorithmic systems may influence the very neural mechanisms that underpin autonomy and social cognition. Children and adolescents, whose brains are undergoing critical periods of plasticity, remain particularly vulnerable to such effects. Addressing these challenges demands an interdisciplinary framework that unites neuroscience, ethics, education, and policy. Proactive research, transparent algorithmic design, and the development of neuroprotective digital environments are essential to ensure that AI supports, rather than subverts, healthy cognitive and moral development. Anticipatory action today may prevent a future in which the architecture of the mind is quietly rewritten by the logic of algorithms.

## Data Availability

All data and materials used in this editorial are derived from publicly accessible sources, including peer-reviewed articles, and referenced scientific literature. Full citations are provided within the text to ensure transparency and facilitate further exploration.
